# Socioecology of the Canine Population in the Province of El Jadida, Morocco

**DOI:** 10.1155/2018/4234791

**Published:** 2018-06-27

**Authors:** K. Bouaddi, A. Bitar, A. Ferssiwi, M. Bouslikhane, A. Fitani, P. P. Mshelbwala, S. W. Audu

**Affiliations:** ^1^Provincial Veterinary Service of El Jadida, Morocco; ^2^Laboratory of Biochemistry, Nutrition and Valorisation of Natural Resources, Faculty of Science, Chouaib Doukkali University, El Jadida, Morocco; ^3^Department of Pathology and Veterinary Public Health, Agronomic and Veterinary Institute Hassan II, Rabat, Morocco; ^4^Private Veterinarian El Jadida, Morocco; ^5^Department of Veterinary Medicine, Faculty of Veterinary Medicine, University of Abuja, Nigeria; ^6^Department of Veterinary Medicine, Ahmadu Bello University, Zaria, Nigeria

## Abstract

Understanding the socioecology of domestic dog populations is essential for effective disease control, especially canine rabies. In Morocco, since 1986, the control efforts and plans put in place by the government have failed to eradicate this disease; this is because the management of the canine population was not taken into account during the establishment of these plans. It is against the background that this study was designed to estimate the dog population and determine its socioecological characteristics, as well as investigate the attitude of the inhabitants towards the dogs. A stratified random sampling was conducted using a structured questionnaire from May to December 2016. A total of 1931 households were interviewed, comprising 27.4% in urban areas and 72.6% in rural areas. A total of 3719 dogs were counted alongside a human population of 11302 for a dog : human ratio of 1 : 2.42 in rural areas and 1 : 46.58 in urban areas. The majority of dogs (92%) in rural areas were not vaccinated against rabies. In urban areas, about 88.5% were vaccinated against rabies. In addition, 78.5% of dogs in rural areas were free roaming, with more than 53% of births being abandoned by their owners, resulting in a large stray and feral dog population and increasing the potential for continued transmission of rabies virus. There was strong association between breed and rabies vaccination (p<0.05) and confinement with body condition score.

## 1. Introduction

Rabies is one of the oldest known infectious diseases, described in the year 2300 BC [1]. In 1885, the first inoculation of a rudimentary vaccine against rabies was carried out by Louis Pasteur, a Parisian researcher, who saved the life of young Joseph Meister [2]. Nevertheless, despite high effective vaccine, rabies remains endemic in many countries in a range of reservoir hosts, with domestic dog as the major reservoir in Africa. Rabies can be transmitted through the saliva of infected animals and can be easily controlled by canine vaccination [3, 4]. It has been estimated that nearly 60,000 people die each year from rabies, with the majority being children and 99% from bites from infected dogs [5–7]. At the same time, the number of anti-rabies treatments per year is estimated by the World Health Organization (WHO) at about 6.5 million, resulting in substantial healthcare costs. Despite numerous efforts to prevent rabies in human and animal populations, this disease remains endemic in developing countries of Africa and Asia [8].

Rabies is still endemic in Morocco with domestic dog as the principal reservoir [9]. In Morocco, rabies still kills about twenty people each year, accounting for almost half of the cases annually recorded in North Africa [10]. Rabies has been linked to a rural lifestyle, with nearly 82% of the approximately 317 annual animal cases being reported in rural areas [9, 11].

Despite canine rabies vaccination efforts, the uncontrolled movements of dogs, particularly their gathering in dump sites in search for food and the extension of cities into rural areas, promote the spread of rabies in the country [12].

In addition to the direct impacts of rabies, there are potential socioeconomic impacts. Morocco is periodically targeted by the media of EU countries because of cases of rabies imported from Morocco, potentially impacting national tourism negatively. For example, out of nine cases of “imported” rabies in France between 2001 and 2011, seven were from Morocco [10]. Through the experiences gained during the implementation of decades-long national control programs, a combination of human and animal factors is believed to contribute to the persistence of rabies in Morocco. These include the behavior of the human population (knowledge, attitudes, and practices), multiplicity of stakeholders in the fight against rabies (including problems with coordination between the services and structures), and the status of the dog, including its ecology and place in the sociocultural context.

Currently, it is generally believed that good knowledge of the local canine ecology is indispensable for the implementation of a rabies control strategy [13–19]. However, canine population data in Morocco are limited, with the last official estimate dating to 1999 [20]. This study was designed to estimate the dog population, determine its characteristics, and evaluate the attitudes of the inhabitants towards dog population, in order to obtain information that will help in planning an efficient rabies control program.

## 2. Materials and Methods

### 2.1. Study Area

**Figure 1:**
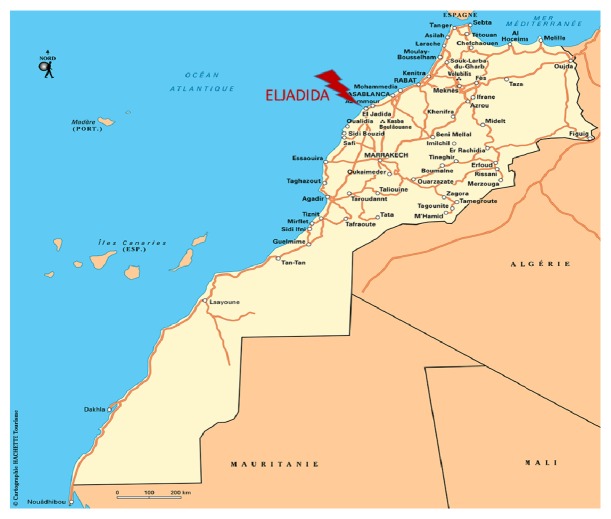
Study area (El Jadida province) Morocco.

The province of El Jadida is one of the richest regions of Morocco due to its climatic diversity, geographical position, and agricultural activity. As part of the region of Casablanca-Settat (Figure 1), the area of action in the province of El Jadida extends over 3 circles, 7 caidats, and 3 municipalities and has 24 municipalities (Figure 3). The total area of the province of El Jadida is 366,821 Hectare(Ha) (useful agricultural area: 281,434 Ha, Bour: 260,336 Ha (93%), irrigated: 21,098 Ha (7%), forest: 18,854 Ha, path and uncultivated: 66,533). Along with agriculture, the majority of farmers practice intensive breeding of sheep and cattle [21].

According to the 2014 General Census of Population and Housing [22], the total population of El Jadida province is 786,716 (40% in urban areas: 312,275 and 50% in rural areas: 474,441).

### 2.2. Survey Method: Method of Survey

**Figure 2:**
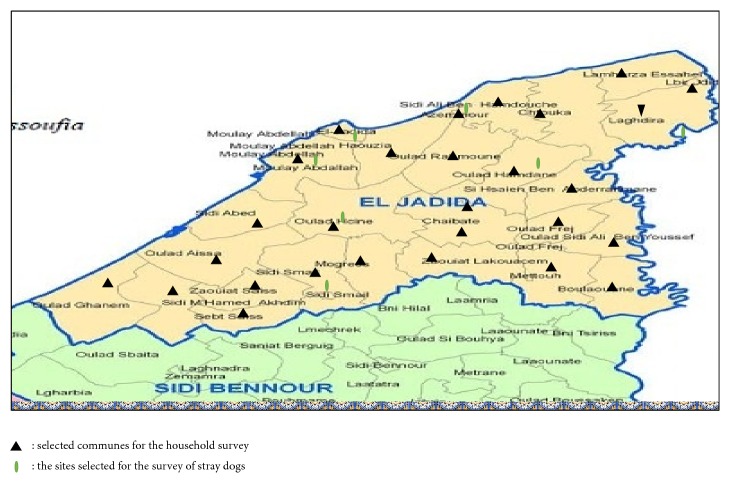
Distribution of sampled places in El Jadida province.

**Figure 3:**
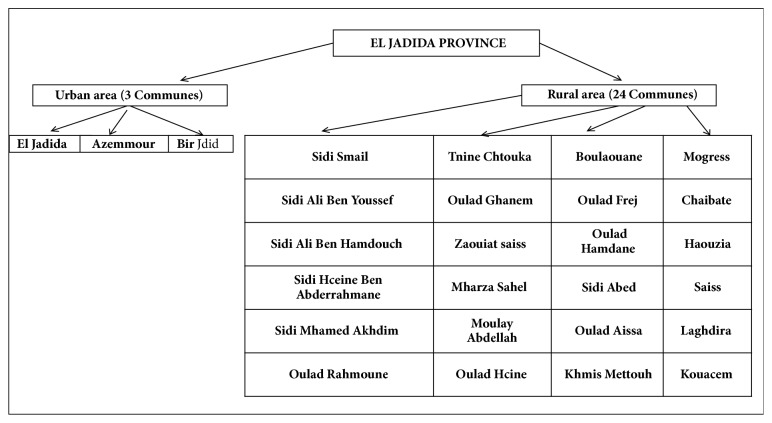
Municipalities concerned by household sampling.

A cross-sectional study was conducted to investigate dog ecology and dog management practices in the province of El Jadida, using structured questionnaires to obtain household- and dog-level data [23]. This included the number of people in the household, control of dogs, number of dogs per household, and individual dog information such as the sex, age, weight, diet, vaccination history, number of litters produced by bitches, and information on the fate of the last litter. The survey was conducted from May to December 2016 in three urban and twenty-four rural municipalities of El Jadida (Figure 2).

### 2.3. Estimate of the Total Canine Population

#### 2.3.1. Estimate of Owned Dogs

The size of the owned dog population was estimated by dividing the number of existing persons in the surveyed households on the number of dogs found in the corresponding households, in order to deduce the dog-to-human ratio. Based on last census figure, which was conducted in the year 2014, the total number of owned dogs in the province of El Jadida was estimated.

#### 2.3.2. Estimation of Free Roaming Dogs

We adopted Beck's method to estimate the number of free roaming dogs [24] (Figure 2). The six locations selected by simple random sampling are areas with a radius of 1km^2^ each located by GPS, belonging to the following municipalities: El Jadida city, Azemmour, Oulad Hamdane, Oulad Hcine, Sidi Smail, and Moulay Abdellah. These are dogs although owned but are allowed to move freely [25].

The method entails making two visits on the 1st and 2nd day and counting the dogs found and marked by photography. The number of stray dogs counted in the selected areas was estimated using the formula: N = Mn / m.

Or

  M is the number of dogs observed for the first time and individually identifiable by a method (photography);
  n is the total number observed the second time;
  m is the number of dogs recognized as previously photographed, which are “re-observed”;
  N is the estimate of the population [13].

#### 2.3.3. Data Processing and Analysis

Data generated was analysed using the statistical packages for social sciences (SPSS) Version 17.0. Data obtained was presented using tables and charts. Chi-square test was used where appropriate to test for association of variables obtained. P values < 0.05 were considered significant.

## 3. Results

### 3.1. Population Size of Owned Dogs

Throughout the study, all households selected by sampling participated fully in the survey, with a very low nonresponse or partial response rate (0% in urban and 0.2% in rural areas). In total, 1931 households were interviewed, inhabited by 11302 people, with 3719 dogs (Table 1).

Based on the census of the Office of the High Commissioner for Planning, held in the year 2014, the population of the inhabitants in the province of El Jadida in urban areas was 312,275 and that of rural was 474,441. Based on an average annual growth rate in the urban environments of 2.62% and 1.18% in rural regions, a total of 207 765 dogs (2909 urban, 204856 rural) were estimated.

### 3.2. Size of the Free Roaming Dog Population

The average density of stray dogs was 12.25 dogs/km^2^ (Table 2), given that the area of the province of El Jadida is 3357,85km2 [26].

### 3.3. Ecological Data on the Studied Dog Population

Dogs less than one year were classified as young, while those above one year were considered adults, based on the information provided by the owner (Table 2). The sex distribution shows the predominance of males (65.47%), with a sex ratio of male to female of the order of 3:1 and 1.87:1 in urban and rural areas, respectively. There was strong association between free roaming dogs in urban and rural areas (P=0.003). The majority of dogs were free roaming in rural areas (78.46%), unlike dogs in urban areas (only 11.54) (Table 3).

**Figure 4:**
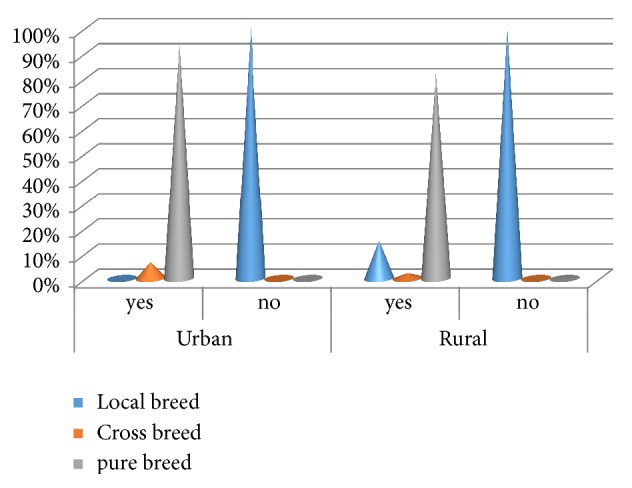
Illustration of the correlation between vaccination and breed.

Most puppies born in rural areas were abandoned (51.4%) to fend for themselves, although this was not the case with urban participant (Table 4). The majority of rural dogs (92.42%) were not vaccinated against rabies (Table 4). There was a strong association between the vaccination status of dogs and the breed, and the majority of pure bred dogs received vaccination in both urban (93.49%) and rural areas (82.73%) (Figure 4).

**Figure 5:**
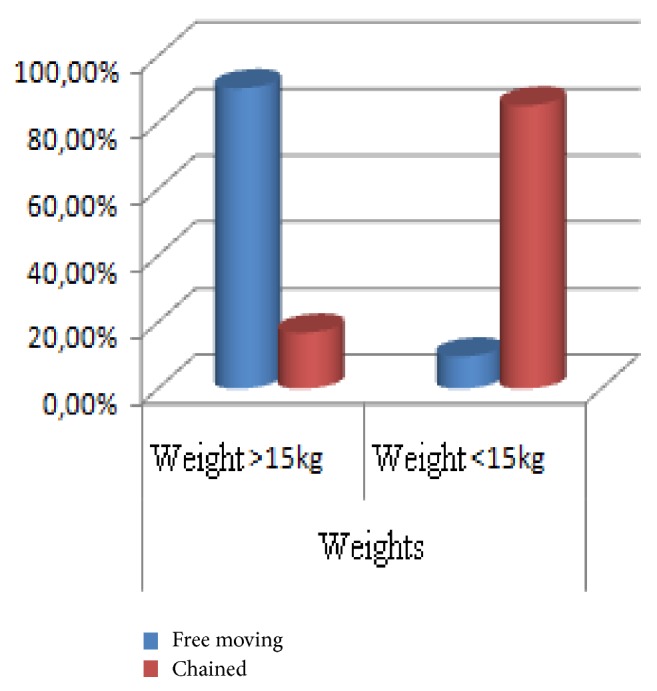
Correlation between weight and lifestyle of dogs.

Also, there was a strong correlation between the weight of dogs (at a distance, based on body condition score) and their way of life in rural areas with free roaming dogs seen to add more weight as opposed to confined and chained dogs (Figure 5).

## 4. Discussion

Population estimation is necessary to develop realistic control plans for dog population management and zoonoses, as well as to monitor the results of these interventions. However, in order to design effective management plans, it is not enough to know the crude size of the populations. Information on the distribution of the dog population, the proportions of feral and owned dogs, management practices, and attitudes of owners are necessary to develop proper interventions that may go a long way in addition to the crude size [12, 27]. This study revealed a high population of dogs in the province of El Jadida (248 898 dogs), with a higher ratio of male dogs in rural areas (1:2.42) as opposed to urban areas (1:46.57). This agrees with the report of Khayli [28] and El Yamani [29] in Morocco and Ratsitorahina et al. [30] in Antananarivo, Madagascar; this is not unconnected to the general belief that male dogs are best in terms of security as well as in monitoring herds in rural region. Increased female dog mortality during pregnancy and puppy rearing may be a reason for the low number of female dogs [19, 31, 32]. Mshelbwala et al. 2013 [33] reported the use of male dog for burial ceremonies in South East Nigeria. The average number of dogs per household in rural areas was 2.61 which is similar to 2:9 in Morocco [32], 1:8 in Kenya, Kitala et al. [34], and 1: 4.5 in Zimbabwe [35], but slightly differs with 1:3.7 in Gwagwalada, Nigeria [19], 1:11 in South Africa [36], and 1:45 for an urban area in Zambia [37].

The majority of dogs in the rural areas (78.46%) were not properly fed by their owners or were only provided with human food remnants, mostly little quantity that is not capable of meeting their daily requirement. This relates to both the low body condition score and body weight of the dogs sampled in this study. Also, some are allowed to roam freely in search for food and shelter, thereby increasing the chances of interactions with humans and potentially increasing dog-dog interactions (including fights) at areas where food may be obtained.

It was estimated that 41,133 dogs although owned are allowed to roam freely. The high number of free roaming dogs is of great public health concern, because of the tendency of such dogs to bite children and adults in that area, as well as increasing the chance for contact with rabid dogs. Previous reports have associated free roaming dogs as sources of nuisance in the society through bites and fecal contamination of the environment [38–41]. The other attitude that aggravates the situation is the abandonment of new puppies born along the main roads. Puppies survive harsh conditions and contribute to the future generation stray and unvaccinated dogs, consequently facilitating the maintenance of rabies virus in the population [19, 32]. Also, the high population of dogs can result in indiscriminate mating which will in turn increase the population further.

Vaccination against rabies remains the key component of rabies control; however, in this current study, only 7.58% of dogs were vaccinated against rabies. This rate is very low compared to the recommendation of the World Health Organization [42] which suggests a vaccination rate of 70% to break the cycle of rabies transmission. The reasons for nonvaccination of dogs are for the most part lack of financial means, lack of awareness of the severity of the disease, and lack of time; this is consistent with previous reports [19, 29, 41]. In addition, despite the free vaccination campaigns carried out annually by the veterinary services of the province of El Jadida in the rural communities, there is low participation of the owners of such dogs; this is because most of their dogs are free roaming and are difficult to restrain and present for vaccination. This is a huge challenge for rabies control program.

In conclusion, the results of this study showed the influence of human factors on the canine population, the nonvaccination of dogs by their owners, the abandonment of newborn puppies, and the encouragement of straying dogs by their owners, all of which increase the population of free roaming dogs and the maintenance of rabies in the area. Human factors are critical and must be taken into account in the management of the dog population and the development of rabies control programs. The most effective way to resolve these human factors is to educate and empower dog owners. Dog owners need to be made aware of the requirements of owning a dog. There is a need for legal mechanisms to empower competent authorities to impose sanctions on irresponsible owners or to take any other necessary action against them.

## Figures and Tables

**Table 1:** Distribution of the number of dogs according to the environment.

Area	Number of households surveyed	Number of people	Number of dogs	Ratio Dog/households	Dog: Human
Urban	529(27.4%)	2422(21.4%)	52(1.4%)	1:10.17	1:46.57

Rural	1402(72.6%)	8880(78.6%)	3667(98.6%)	2.61:1	1:2.42

*Total*	*1931(100%)*	*11302(100%)*	*3719(100%)*	*-*	*-*

**Table 2:** Estimation of free roaming dogs and their density.

Area	Commune	M	n	M	Σ (Mn)	Mn/m	Density (dogs/km^2^)
Urban	El Jadida	5	6	3	30	10	10
	Azemmour	9	11	5	99	19,8	19,8

Rural	Moulay Abdellah	12	13	10	156	15,6	15,6
	Sidi Smail	8	9	8	72	9	9
	Oulad Hamdane	9	8	7	72	10,28	10,28
	Oulad Hcine	7	7	6	49	8,16	8,16

**Table 3:** Ecological characteristics of dogs surveyed.

**Character**		**Urban (**%**)**	**Rural (**%**)**
**Sex**	**Male**	39(75%)	2396(65,3%)
	**Female**	13(25%)	1271(34,7%)

** Class**	**Young**	10 (19,2%)	818 (22,3%)
	**Adult**	42(80,8%)	2849 (77,7%)

**Way of life**	**On chain**	46 (88,5%)	790 (21,5%)
	**Move freely**	6 (11,5%)	2877 (78,5%)

**Purpose**	**Guarding**	34(65,4%)	3525(96,1%)
	**Hunt**	3(5,8%)	140(3,8%)
	**Company**	15(28,8%)	13(0,3%)

**Dog Breed**	**Local**	6 (11,5%)	3519 (96%)
	**Cross-breed**	3 (5,8%)	7 (0,2%)
	**Pure breed**	43 (82,7%)	141 (3,8%)

**Table 4:** Owner's attitude towards the feeding and vaccination.

**Characters**		**Urban (**%**)**	**Rural (**%**)**
**Anti-rabies vaccination in life time**	**Yes**	46 (88,5%)	278 (7,6%)
	**No**	6 (11,5%)	3389 (92,4%)

**Dog food**	**Family leftover**	24 (46,2%)	3568 (97,3%)
	**Commercial dog food**	28 (53,9%)	80 (2,2%)
	**public dumps**	0 (0%)	19 (0,5%)

**Fate of puppies**	**Keep**	0(0%)	152 (14,7%)
	**Abandoned**	0(0%)	531 (51,4%)
	**Giving**	2 (25%)	338 (32,7%)
	**Sold**	6 (75%)	12 (1,16%)

## Data Availability

The data used to support the findings of this study are available from the corresponding author upon request.
